# Exploiting Blood Transport Proteins as Carborane Supramolecular Vehicles for Boron Neutron Capture Therapy

**DOI:** 10.3390/nano13111770

**Published:** 2023-05-31

**Authors:** Tainah Dorina Marforio, Edoardo Jun Mattioli, Francesco Zerbetto, Matteo Calvaresi

**Affiliations:** Dipartimento di Chimica “Giacomo Ciamician”, Alma Mater Studiorum-Università di Bologna, Via Francesco Selmi 2, 40126 Bologna, Italy; edoardojun.mattioli2@unibo.it (E.J.M.); francesco.zerbetto@unibo.it (F.Z.)

**Keywords:** carborane, boron neutron capture therapy (BNCT), virtual screening, docking, reverse docking, MD simulations, MM-GBSA, plasma proteins

## Abstract

Carboranes are promising agents for applications in boron neutron capture therapy (BNCT), but their hydrophobicity prevents their use in physiological environments. Here, by using reverse docking and molecular dynamics (MD) simulations, we identified blood transport proteins as candidate carriers of carboranes. Hemoglobin showed a higher binding affinity for carboranes than transthyretin and human serum albumin (HSA), which are well-known carborane-binding proteins. Myoglobin, ceruloplasmin, sex hormone-binding protein, lactoferrin, plasma retinol-binding protein, thyroxine-binding globulin, corticosteroid-binding globulin and afamin have a binding affinity comparable to transthyretin/HSA. The carborane@protein complexes are stable in water and characterized by favorable binding energy. The driving force in the carborane binding is represented by the formation of hydrophobic interactions with aliphatic amino acids and BH-π and *C*H-π interactions with aromatic amino acids. Dihydrogen bonds, classical hydrogen bonds and surfactant-like interactions also assist the binding. These results (i) identify the plasma proteins responsible for binding carborane upon their intravenous administration, and (ii) suggest an innovative formulation for carboranes based on the formation of a carborane@protein complex prior to the administration.

## 1. Introduction

Boron neutron capture therapy (BNCT) is a non-invasive binary approach for the treatment of difficult-to-cure tumors such as glioblastoma multiforme or head and neck cancers [1,2,3,4,5,6]. The effectiveness of BNCT is based on the adequate and selective accumulation of ^10^B in the tumor tissue (approximately 10^9^ atoms/cell), followed by irradiation with a thermal neutron beam. The fission reaction that occurs upon the neutron beam irradiation on the boron atom (^10^B[n,α] ^7^Li) produces a high linear energy transfer (LET) alpha particle (^4^He), a recoiled lithium nucleus (^7^Li) and γ radiation (0.48 MeV). The alpha particles, which have a LET in the range of 50–230 keV/μm, also possess a very short travel distance, causing their energy to be released within the diameter of the cell (Bragg’s peak). Thus, BNCT is potentially highly selective, since only the cells that have accumulated enough boron are destroyed by the radiation. Low uptake and toxicity in healthy cells and persistence in the malignant tissues are the requirements for effective BNCT agents. The first boron-containing compounds employed in BNCT were based on boric acids and their derivatives (first-generation compounds). In the 1980s, concomitantly with the improvement of neutron beam sources, BPA (boronphenylalanine) and BSH (sodium borocaptate) were extensively employed in BNCT, and were authorized for clinical trials [7].

Nowadays, third-generation agents are emerging as potential drugs for BNCT clinical applications, such as boronated natural and non-natural amino acids [8,9], boronated drugs [10], nucleosides [11,12], porphyrines [13,14,15], antibodies [16] and carbohydrates [17]. Boron-based nanomaterials [18,19,20,21], such as polymers [22], liposomes [23,24,25,26], boron nitride [20,27] and other types of nanoparticles, were also developed [28,29,30,31,32,33].

Icosahedral carborane (1,2-C2B10H12) [4,34,35], which belongs to the family of closo-carboranes, promises to be a good boron agent candidate in BNCT because of (i) the presence of 10 boron atoms per molecule, (ii) its abiotic nature, which prevents metabolism and resistance by the living organism, and (iii) its chemical structure, which provides the cage with a strong hydrophobic character (3D aromaticity of the carborane cage) [36,37] facilitating the crossing of hydrophobic membranes, such as the cellular membrane [38,39,40,41,42]. On the other hand, the hydrophobicity of carboranes hampers their direct administration, being insoluble in blood; for their administration, organic solvents [43,44] or co-formulants [13,45,46,47] are usually used. The formation of complexes between the blood transport proteins and the carborane governs its cellular uptake and biodistribution. Human serum albumin (HSA) is one of the best biocompatible excipients for the solubilization and de-aggregation of cobalt bis(dicarbollide) derivatives in water [48]. Metallacarboranes bind to the surface of HSA [49] and hydrophobic interactions govern the binding because the binding affinity correlates with the lipophilicity of the metallacarborane derivatives [50].

The design of carriers able to transport carboranes in a physiological environment is a pressing task for BNCT’s transition from an experimental modality to a widely accepted clinical approach.

The idea of employing proteins as hydrophilic carriers for hydrophobic drugs represents a novel and smart strategy to generate a biocompatible and water-soluble system for nanomedical and theranostic approaches [51,52,53,54]. Proteins are ideal supramolecular hosts for carrying and delivering carboranes since they meet the requirements of biocompatibility and water solubility and are naturally recognized by cells. Carborane has already demonstrated its ability to interact strongly with protein-binding sites; in fact, carborane derivatives have been used as inhibitors for many proteins such as HIV protease, human carbonic anhydrase, estrogen and androgen receptor, dihydrofolate reductase, translocator protein, retinoic acid receptor and retinoid X receptors, transthyretin, cyclooxygenase-2 and others [55,56].

**Scheme 1:**
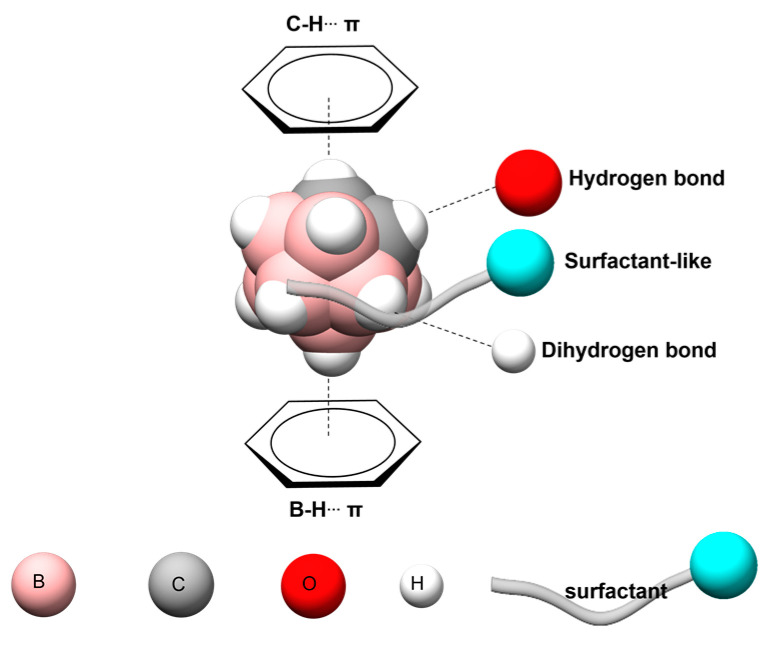
Representation of the possible intermolecular interactions that can be generated between carborane and the protein-binding pockets.

Carboranes can generate a variety of intermolecular interactions (Scheme 1) with amino acids due to the presence of both acidic C-H and hydridic B-H groups, i.e., those bound to carbon and boron atoms, respectively: (i) “classical” hydrogen bonds (HB) between the acidic C-H groups of the carborane and a hydrogen bond acceptor (C–H···X), (ii) dihydrogen bonds (HH) between the hydridic B-H groups of the cage and a hydrogen bond donor (B–H···H–X), (iii) CH^…^π interactions between the acidic C-H groups of the carborane and aromatic residues, (iv) BH^…^π interactions between the hydridic B-H groups of the carborane and aromatic groups [37,57,58,59,60,61] and (vi) surfactant-like interactions where the non-polar portion of an amphiphilic molecule interacts with the hydrophobic cage of the carborane while the polar part protrudes in the solvent.

Proteins, acting as Trojan horses [56,62,63], can also promote the accumulation of boron in the tumor tissue by taking advantage of the enhanced permeability and retention (EPR) effect and the tumor-targeting activity of some specific proteins [64,65,66]. 

Among the human proteome, blood transport proteins are the natural carriers of endogenous and exogenous hydrophobic molecules in the bloodstream [67]. Blood transport proteins have well-defined binding pockets for the transport of non-soluble compounds [67]. Human serum albumin (HSA), the most abundant protein in blood, and α-fetoprotein (AFP) and afamin are responsible for the binding of fatty acids, exogenous drugs and hormones [67]; vitamin D-binding protein (DBP), thyroxine-binding globulin (TBG), transthyretin (TTR), corticosteroid-binding globulin (CBG), sex hormone-binding globulin (SHBG) and plasma retinol-binding protein (RBP) carry hormones, steroids and vitamins in plasma [67]. Other proteins, such as hemoglobin (Hb), myoglobin (Mb), hemopexin (HPX) and haptoglobin (Hp), are heme-binding proteins and they are generally responsible for the transport of oxygen and carbon dioxide [67], while cerulosplasmin (CP), serotransferrin (TF), lactotransferrin (LF) and ferritin (FT) are involved in iron/copper transport and storage [67].

Carboranes are highly hydrophobic [36,37], and their transport in blood as pristine moieties is hardly possible. The notion of using plasma proteins as potential carriers of hydrophobic species is rather straightforward. In addition, the different chemical moieties present on the protein surface can be chemically functionalized with (i) targeting tags able to improve both the cell selectivity and promote the selective uptake of the carborane@protein hybrid in cancer therapy and (ii) imaging tags to create innovative, protein-based theranostic platforms [68].

Some of the blood transport plasma proteins can also be uptaken by cancer cells via active targeting: cancer cells can selectively accumulate human serum albumin and transferrin because of the high expression levels of albumin-binding proteins (gp60 and SPARC) and transferrin receptors. BNCT was clinically approved for head and neck cancers; it is well known that many head and neck cancers overexpress SPARC and transferrin receptors [69,70]. So, the use of HSA and transferrin as carborane carriers can be potentially exploited for the development of boron neutron capture targeted therapy. 

HSA and transferrin are also effective carriers for delivery across the blood–brain barrier, and the dispersion of carborane by these proteins can be an opportunity for the application of BNCT to brain cancers [71].

Therefore, in this work, a reverse docking approach was used to investigate the interaction of blood plasma with carborane. Molecular dynamics (MD) simulations, followed by molecular mechanics/generalized Born surface area (MM-GBSA) analysis, quantitatively evaluated the affinity of carborane to blood transport proteins. This approach allows for (i) the identification of the most promising proteins to act as carriers for carborane in blood, (ii) the characterization of the carborane-binding pocket(s) and (iii) the determination of the nature of the non-covalent interactions between the amino acids of the protein and the ligand. 

## 2. Materials and Methods

### 2.1. Blood Transport Protein Structural Database 

Blood transport proteins were identified following the classification of Schaller [67]. When the 3D structures of the proteins are experimentally available, they were downloaded from the Protein Data Bank [72]. When multiple PDBs were available, a representative structure was identified, using as selecting criteria the completeness of the sequence, the absence of mutations and the highest resolution. These structures, reported in Table 1, were cleaned of their co-crystallized ligands, ions and water molecules. When the 3D structure was not experimentally available, as in the case of hemopexin, the computed structure model (CSM) was used [73]. 

### 2.2. Docking and Refinement

The *ortho*-carborane structure was used as a representative of the closo-carboranes, due to its higher stability compared to meta- and para-carboranes. Docking calculations were carried out using the ortho-carborane structure and the blood transport proteins structural database, using the PatchDock algorithm [77], which is a valid tool for generating poses for rigid and spherical ligand molecules such as fullerenes [78,79,80,81,82,83,84,85,86], carbon nanotubes [87,88,89,90] and carboranes [55]. All the carborane@protein complexes obtained as poses of the docking procedure were then refined by minimization and MM-GBSA calculations. The first 50 poses, sorted by binding affinity, were further refined by 1 ns MD simulation followed by MM-GBSA calculations. For the complex of each blood transport protein, characterized by the highest affinity, 100 ns of MD simulations were carried out. In the case of human serum albumin (HSA), a refinement of 100 ns was carried out for all the possible binding pockets.

### 2.3. MD Simulations

The FF14SB force field [91], as implemented in Amber, was used to describe the proteins, while an ad hoc force field was used for the ortho-carborane, combining the GAFF force field with the parameters developed by Sarosi et al. [92]. In all simulations, the carborane@protein complexes were solvated by explicit water molecules (TIP3P model) and Na^+^ and Cl^−^ counterions were added to neutralize the total charge of the system. Periodic boundary condition (PBC) and the particle mesh Ewald summations, with a cut-off radius of 10.0 Å, were used during all the simulations. RMSD values of carborane@protein complexes (see Figures S1–S15) were calculated using CPPTRAJ [93].

### 2.4. Molecular Mechanics/Generalized Born Surface Area (MM-GBSA) Calculations

From each MD trajectory, 1000 frames were extracted by CPPTRAJ [93] and employed as input for the MM-GBSA analysis [94]. The MM-GBSA analysis was carried out using the MM-PBSA.py module, considering an infinite cut-off in the calculation of the electrostatic and van der Waals (vdW) interactions. The generalized Born (GB) model developed by Hawkins and coworkers (GB^HCT^) was used to compute the polar solvation term [95,96], whereas the non-polar solvation term was determined using solvent-accessible, surface-area-dependent terms. A fingerprint analysis, based on the decomposition of the total binding energy on a per-residue basis, offers the possibility to identify the protein residues more responsible for the binding of the ligand.

## 3. Results and Discussion

### 3.1. Identification of Blood Transport Proteins as Carriers for Carborane by Virtual Screening 

**Figure 1:**
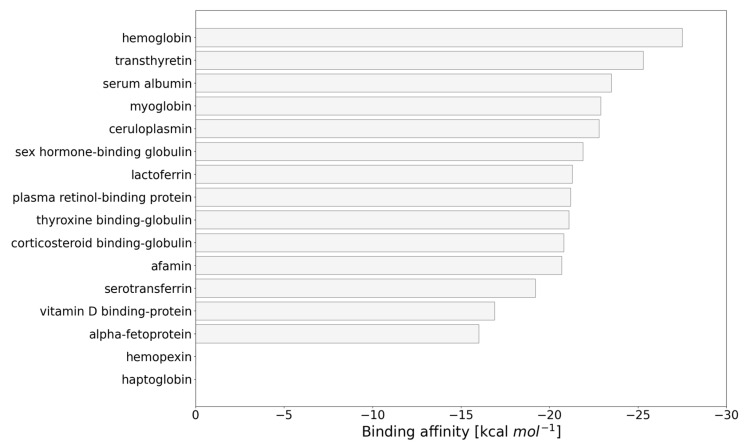
Binding affinity (kcal mol^−1^) of the blood transport proteins for the carborane, sorted by the affinity for the ligand.

A protocol of reverse docking was used to identify the propensity of the blood transport proteins to bind carborane. Figure 1 shows the strength of the interaction between blood transport proteins and carboranes, as calculated by the MD simulation followed by MM-GBSA analysis. The binding between carborane derivatives and HSA [18,49,97,98,99,100] and transthyretin [101] has already been experimentally demonstrated. 

Hemoglobin has a stronger binding with carborane (−27.5 kcal mol^−1^) with respect to transthyretin (−25.3 kcal mol^−1^) and serum albumin (−23.5 kcal mol^−1^). Eight other proteins, i.e., myoglobin (−22.9 kcal mol^−1^), ceruloplasmin (−22.9 kcal mol^−1^), sex hormone-binding protein (−22.5 kcal mol^−1^), lactoferrin (−21.3 kcal mol^−1^), plasma retinol-binding protein(−21.2 kcal mol^−1^), thyroxine-binding globulin (−21,1 kcal mol^−1^), corticosteroid-binding globulin (−20.8 kcal mol^−1^) and afamin (−20.7 kcal mol^−1^), show a binding affinity comparable (higher than 20 kcal mol^−1^) to transthyretin and HSA, and can potentially bind carborane.

### 3.2. Carborane@HSA

Serum albumin (HSA) is the most abundant protein in the blood. HSA is responsible for the binding of fatty acids, exogenous drugs and hormones [67]. HSA has already demonstrated its ability to bind carborane derivatives [18,49,97,98,99,100].

The interactions of the carborane moieties with HSA were ascribed to the formation of hydrophobic interactions between the lipophilic cage of the carborane and the non-polar binding pockets of HSA. 

**Figure 2:**
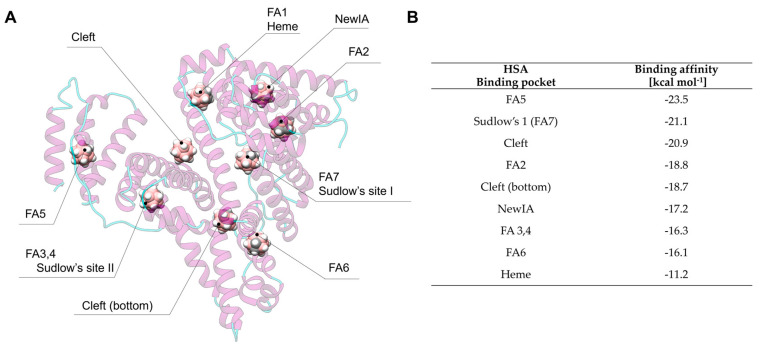
(**A**) The most probable carborane-binding sites in HSA; (**B**) the binding affinity of carborane for the HSA pockets computed by MM−GBSA approach after 100 ns of MD simulation in explicit water.

Nevertheless, to our knowledge, the structural characterization of the carborane@HSA complex has never been reported. Following the proposed docking approach, we identified the most probable binding sites for carborane in HSA. The carborane occupies all the binding pockets that are specific for fatty acids (FA1–7), and the cleft site (Figure 2). The highly hydrophobic nature of the carborane explains its tendency to bind in the HSA pockets where hydrophobic molecules, such as warfarine (Sudlow’s 1), ibuprofen (FA6), iodipamide (cleft), oxyphenbutazone (FA5) and diazepam (FA3,4), usually bind to HSA. Two additional binding pockets for the carborane were also identified. The binding affinity computed for the carborane in each of the nine pockets is reported in Figure 2B. 

**Figure 3:**
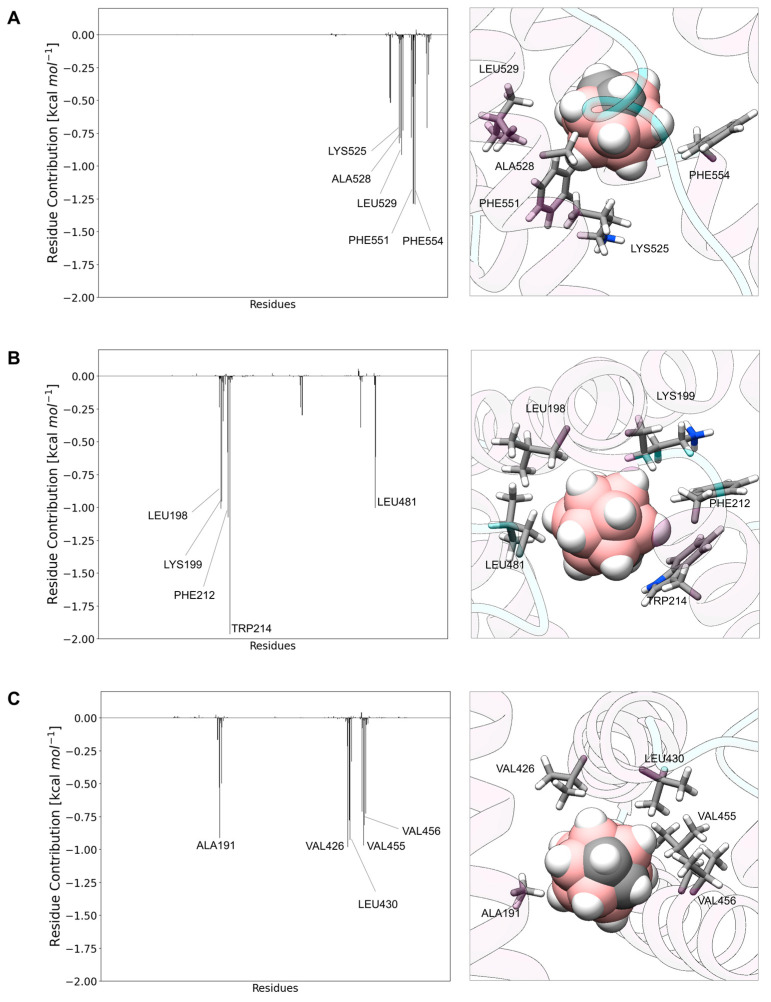
Fingerprint analysis and 3D representation of the interactions between carborane and HSA in the (**A**) FA5-binding pocket, (**B**) Sudlow’s site I and (**C**) cleft region.

After 100 ns MD simulations, the three most favorable binding pockets were found to be FA5 (−23.5 kcal mol^−1^), Sudlow’s I (−21.1 kcal mol^−1^) and the cleft region (−20.9 kcal mol^−1^). Our results suggest that FA5, located in domain III of HSA, is the principal site for carborane binding in HSA. Carborane interacts with Phe554 (−1.3 kcal mol^−1^) and Phe551 (−1.3 kcal mol^−1^) by BH-π and CH-π interactions (Figure 3A). Leu529 (−0.9 kcal mol^−1^) and Ala528 (−0.8 kcal mol^−1^) give non-polar interactions with the carborane, while Lys525 (−0.8 kcal mol^−1^) is characterized by a surfactant-like interaction: the aliphatic side-chain of Lys binds the hydrophobic surface of the carborane, while its hydrophilic head interacts with water. 

At Sudlow’s site I (Figure 3B), the carborane cage interacts by BH-π interactions with Trp214 (−2.0 kcal mol^−1^) and with Phe212 (−1.1 kcal mol^−1^). Leu198 (−1.0 kcal mol^−1^) and Leu481 (−1.0 kcal mol^−1^) are other residues responsible for the binding via hydrophobic interactions. Also in this binding pocket, similarly to Lys525 in FA5, Lys199 (−1.0 kcal mol^−1^) gives surfactant-like interactions.

When carborane is located in the cleft (Figure 3C), only non-polar amino acids are responsible for the binding, specifically Val426 (−1.0 kcal mol^−1^), Val455 (−1.0 kcal mol^−1^), Leu430 (−0.9 kcal mol^−1^), Ala191 (−0.9 kcal mol^−1^) and Val456 (−0.8 kcal mol^−1^).

### 3.3. Carborane@transthyretin 

**Figure 4:**
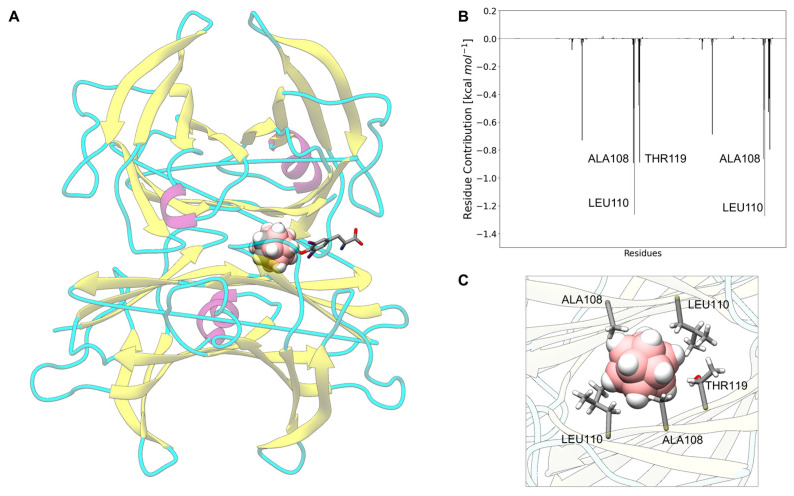
(**A**) A 3D representation of the carborane@transthyretin superimposed with T_4_ (PDB ID 5CR1). (**B**) *Per*-residue decomposition energy. (**C**) Close-up of the most interacting protein residues with the ligand.

Transthyretin (TTR) is a homotetramer, responsible for carrying the thyroid hormone thyroxine (T_4_) [67]. TTR also acts as a carrier of retinol (vitamin A) through its association with retinol-binding protein (RBP) [67]. TTR is a 55 kDa protein and it comprises four subunits of 127 amino acids each [67]. The interface between two dimers creates the two binding pockets for T_4_. Less than 1% of TTR’s T_4_-binding sites are occupied in blood. The docking and the MD simulations reveal that carborane occupies the T_4_-binding site (Figure 4A), as also suggested by experimental studies [101]. In this binding pocket (Figure 4B,C), aliphatic amino acids such as the two Leu110 (−1.3 kcal mol^−1^) and the two Ala108 (−0.9 kcal mol^−1^) give strong hydrophobic interactions with the carborane. The hydroxyl side-chain of Thr119 (−0.9 kcal mol^−1^) is involved in dihydrogen bonds with the hydridic B-H groups of the cage. 

### 3.4. Carborane@hemoglobin 

Hemoglobin (Hb) is a metalloprotein responsible for the transport of oxygen to the tissues. Hb (64 kDa) comprises four subunits, namely α and β chains of 141 and 146 residues, respectively, that pack into a tetramer α_2_β_2_ [67]. The four subunits interact via non-covalent interactions, and each chain contains a heme prosthetic group (i.e., iron protoporphyrin IX) which is responsible for the binding of molecular oxygen. 

**Figure 5:**
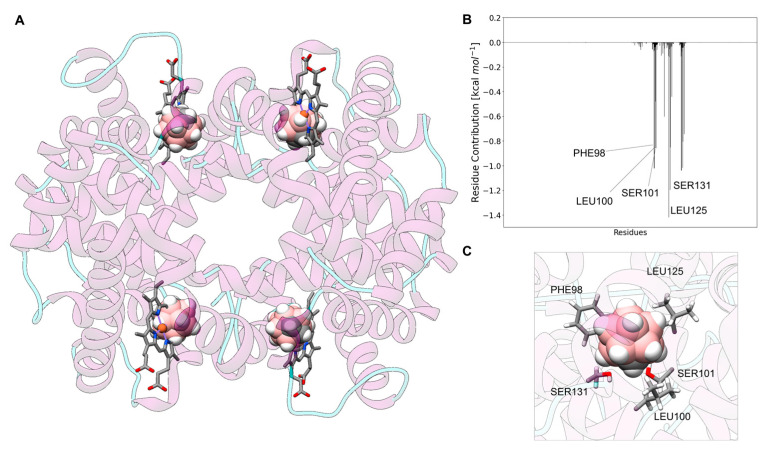
(**A**) A 3D representation of the carborane@hemoglobin superimposed with heme (PDB ID 5HY8). (**B**) *Per*-residue decomposition energy. (**C**) Close-up of the most interacting protein residues with the ligand.

Carboranes occupy the heme-binding pockets of Hb (Figure 5A). This is not surprising since the heme-binding pockets in proteins are usually characterized by the presence of hydrophobic/aromatic residues responsible for the binding of the hydrophobic portions of the heme group.

The fingerprint analysis (Figure 5B,C) suggests that Phe98, Leu100 and Leu125 are the residues that mostly contribute to the stabilization of the carborane inside the heme-binding pocket: Phe98 (−1.4 kcal mol^−1^) by BH-*π* interactions, and Leu100 (−1.2 kcal mol^−1^) and Leu125 (−1.2 kcal mol^−1^) by hydrophobic contacts. Two polar amino acids, i.e., Ser131 (−1.2 kcal mol^−1^) and Ser101 (−1.1 kcal mol^−1^), are also important for the binding, engaging dihydrogen bonds with the carborane cage.

### 3.5. Carborane@myoglobin 

Myoglobin (Mb) is formed by a single polypeptide chain, folded into a globular shape. Mb shares almost identical secondary and tertiary structures with the α and β subunits of Hb and consequently binds only one heme group [67].

**Figure 6:**
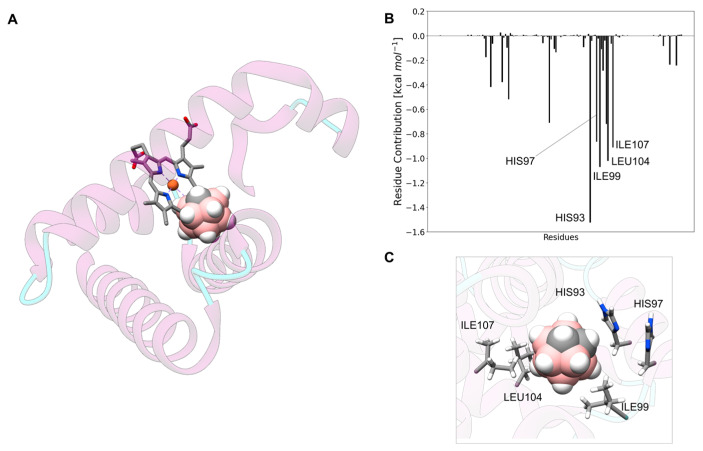
(**A**) A 3D representation of the carborane@myoglobin superimposed with the heme (PDB ID 3RGK). (**B**) Per-residue decomposition energy. (**C**) Close-up of the most interacting protein residues with the ligand.

Mb is responsible for oxygen transport and storage in muscles [67]. In analogy to Hb, our in silico approach suggested that carborane occupies the binding pocket of the heme group of Mb (Figure 6A). His93 (−1.5 kcal mol^−1^) and His97 (−0.9 kcal mol^−1^) interact with the carborane cage by BH-*π* and *C*H-π interactions. Other three non-polar residues are responsible for the formation of the carborane@myoglobin complex via hydrophobic interactions, namely Ile99 (−1.1 kcal mol^−1^), Leu104 (−1.0 kcal mol^−1^) and Ile107 (−0.9 kcal mol^−1^). The fingerprint analysis and the close-up of the most interacting residues are reported in Figure 6B,C, respectively.

Removing the heme group [50], it is possible to use myoglobin as a drug delivery system/supramolecular host for carborane.

### 3.6. Carborane@cerulosplamin 

**Figure 7:**
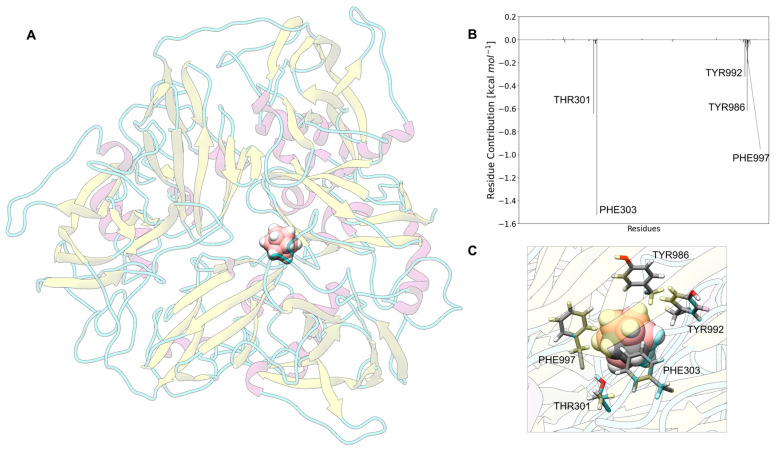
(**A**) A 3D representation of the ortho-carborane@cerulosplamin. (**B**) Per-residue decomposition energy. (**C**) Close-up of the most inter-acting protein residues with the ligand.

Ceruloplasmin (CP) is the most important copper-carrying protein in the blood and plays an important role in iron metabolism [67]. Ceruloplasmin is also able to prevent oxidative damage to biological systems (proteins, DNA and lipids) because of its antioxidant activity. CP has a molecular weight of ~132 kDa and is composed by a single peptide chain; in physiological conditions, it is a trimer [67]. Carborane binds CP at the interface of the three domains (Figure 7A), and the formation of the complex is stabilized by the formation of a set of BH-π and *C*H-π interactions with aromatic residues composing the binding pocket (Figure 7B,C), namely Phe303 (−1.5 kcal mol^−1^), Tyr992 (−1.1 kcal mol^−1^), Phe997 (−1.1 kcal mol^−1^) and Tyr986 (−0.8 kcal mol^−1^). The hydroxyl group of Thr301 (−0.7 kcal mol^−1^), instead, generates a dihydrogen bond. 

### 3.7. Carborane@sex Hormone-Binding Globulin 

**Figure 8:**
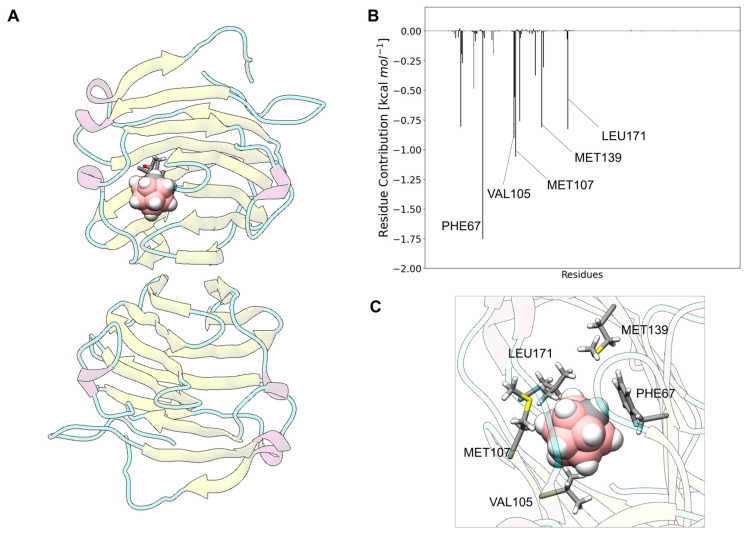
(**A**) A 3D representation of the carborane@sex hormone-binding globulin superimposed with dihydrosterone (PDB ID 1D2S) (**B**) *Per*-residue decomposition energy. (**C**) Close-up of the most interacting protein residues with the ligand.

Sex hormone-binding globulin (SHBG) is a homodimeric glycoprotein responsible for carrying testosterone, dihydrotestosterone androgens and estradiol in the bloodstream [67]. It is produced by the liver. SHBG regulates the level of these hormones, keeping their concentration stable and preventing them from being broken down too quickly. Testosterone generally binds at the N-terminal domain and estrogen at the C-terminal portion of SHBG. The location of carborane in the protein is depicted in Figure 8A, indicating that the carborane is located in the binding pocket of dihydrosterone. At this binding site (Figure 8B,C), the carborane interacts with Phe67 (−1.8 kcal mol^−1^) by BH-*π* and *C*H-π interactions and with the hydrophobic side-chains of Val105 (−0.9 kcal mol^−1^), Met107 (−1.1 kcal mol^−1^), Met139 (−0.8 kcal mol^−1^) and Leu171 (−0.8 kcal mol^−1^). 

### 3.8. Carborane@lactotransferrin 

**Figure 9:**
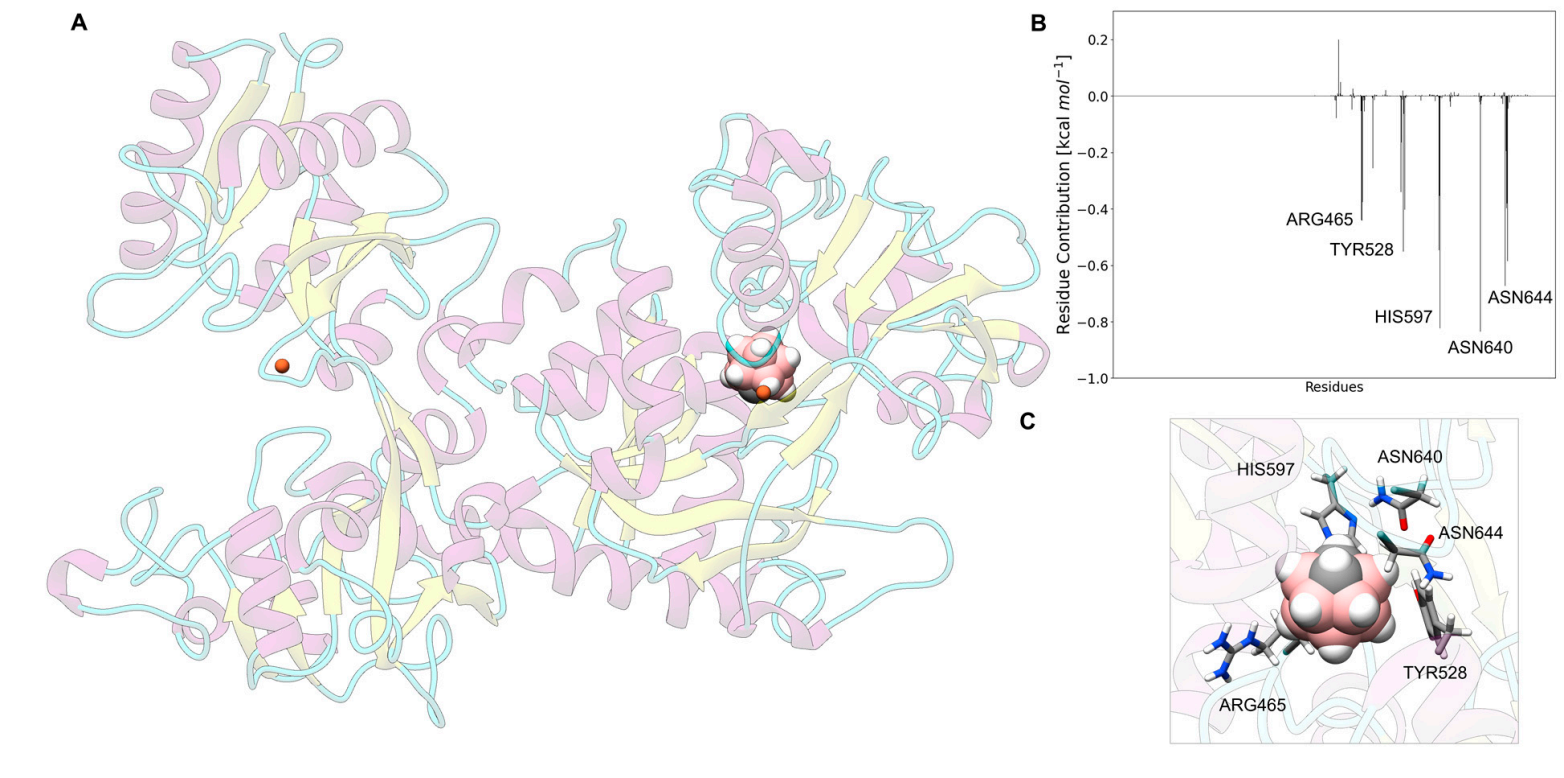
(**A**) A 3D representation of the carborane@lactotransferrin superimposed with the two Fe(III) (PDB ID 1B0L). (**B**) Per-residue decomposition energy. (**C**) Close-up of the most interacting protein residues with the ligand.

Transferrins (LT) are glycoproteins found in mammal fluids, such as blood (seotransferrin) and milk (lactotransferrin) [67]. Even if it is not a heme protein, transferrin is responsible for the binding of two iron atoms, their transport in the body and the regulation of the iron metabolism [67]. The residues involved in Fe(III) binding are also responsible for the recognition of the carborane cage (Figure 9A). In particular, as depicted in Figure 9B,C, the aromatic imidazole of Hie597 (−0.8 kcal mol^−1^) and the phenol ring of Tyr528 (−0.8 kcal mol^−1^) give BH-*π* and *C*H-π interactions with the aromatic cage of the carborane. Arg465 (−0.5 kcal mol^−1^) interacts with its guanidinium group via cation-*π* and BH-*π* interactions, while two asparagine residues, Asn640 (−0.7 kcal mol^−1^) and Asn644 (−0.6 kcal mol^−1^), are involved in the dihydrogen bond with the carborane cage. 

### 3.9. Carborane@plasma Retinol-Binding Protein 

**Figure 10:**
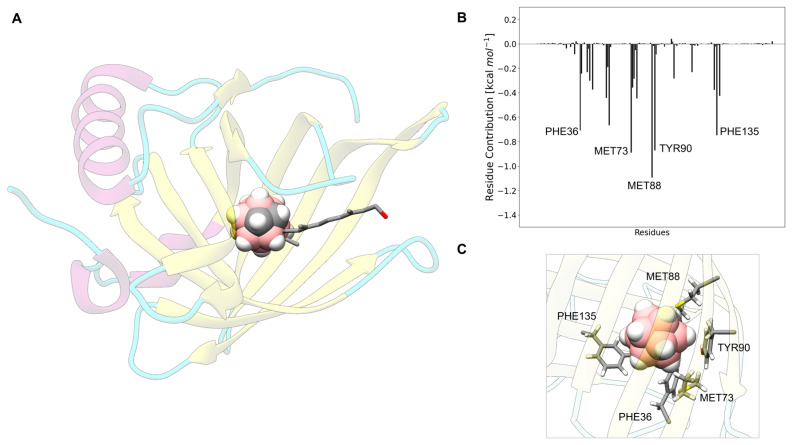
(**A**) A 3D representation of the carborane@plasma retinol-binding protein superimposed with retinol (PDB ID 5NU7). (**B**) Per-residue decomposition energy. (**C**) Close-up of the most interacting protein residues with the ligand.

Plasma retinol-binding protein (RBP) is a single-chain protein of ~180 amino acids. RBP carries retinol (vitamin A) in blood, binding it in a hydrophobic pocket near the center of the protein [67]. Vitamin A is crucial for vision, immune function and cellular growth and differentiation; therefore, adequate levels of RBP are essential for the correct delivery of vitamin A in the body. Carborane occupies the retinol-binding pocket in RBP (Figure 10A); here, two methionine residues, Met88 (−1.1 kcal mol^−1^) and Met73 (−0.9 kcal mol^−1^), engage hydrophobic interactions with the carborane, while three aromatic amino acids, Tyr90 (−0.9 kcal mol^−1^), Phe135 (−0.7 kcal mol^−1^) and Phe36 (−0.7 kcal mol^−1^), give BH-*π* and *C*H-π interactions with the aromatic cage of the carborane (Figure 10B,C).

### 3.10. Carborane@thyroxine-Binding Globulin

**Figure 11:**
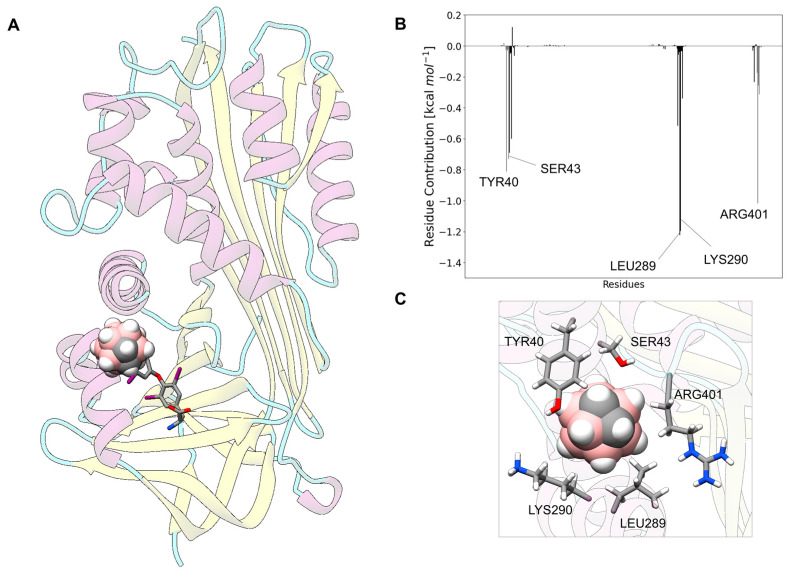
(**A**) A 3D representation of the ortho-carborane@thyroxine-binding protein superimposed with T_4_ (PDB ID 2XN6). (**B**) Per-residue decomposition energy. (**C**) Close-up of the most interacting protein residues with the ligand.

Thyroxine-binding globulin (TBG) regulates the binding of thyroxine (T_4_) and triiodothyronine (T_3_) [67], together with transthyretin and serum albumin. TBG has only one binding site for the thyroid hormones and has a molecular mass of ~54 kDa [67]. Carborane binds TBG in the T_4_-binding pocket (Figure 11A) superimposing to one of the aromatic groups of the endogenous ligand. At this binding site (Figure 11B,C), Leu289 (−1.2 kcal mol^−1^) gives hydrophobic interactions with the carborane. Two charged residues, Lys290 (−1.2 kcal mol^−1^) and Arg401 (−1.0 kcal mol^−1^), interact with the carborane cage with their aliphatic side chains via surfactant-like interactions. The aromatic Tyr40, instead, arranges its phenol group to give sandwich-like BH-*π* and *C*H-π interactions (−0.8 kcal mol^−1^) with the carborane, while the hydroxyl portion of Ser43 (−0.7 kcal mol^−1^) engages a dihydrogen bond with the hydridic B-H groups of the ligand. 

### 3.11. Carborane@corticosteroid-Binding Globulin 

**Figure 12:**
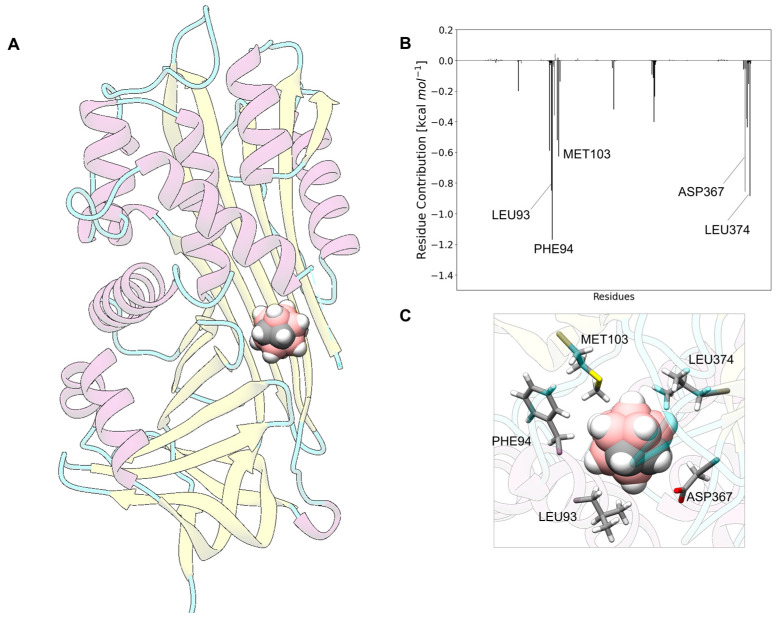
(**A**) A 3D representation of the ortho-carborane@corticosteroid-binding protein. (**B**) Per-residue decomposition energy. (**C**) Close-up of the most interacting protein residues with the ligand.

Glucocorticoids and hormones, such as cortisol and progesterone, are mainly carried in the blood by corticosteroid-binding globulin (CBG), a member of the SERPIN (serine protease inhibitor) family [67]. CBG is a single-chain protein of 383 amino acids with a weight of ~52 kDa. The carborane binds in a pocket (Figure 12A–C) characterized by BH-*π* and *C*H-π interactions with Phe94 (−1.2 kcal mol^−1^) and hydrophobic interactions with Leu93 and Leu374 (−0.9 kcal mol^−1^). Very interestingly, here, a new type of interaction appears, i.e., a hydrogen bond with Asp367 (−0.9 kcal mol^−1^). In fact, the negatively charged carboxylate group of Asp389 interacts with the acidic C-H groups of the carborane cage.

### 3.12. Carborane@afamin

**Figure 13:**
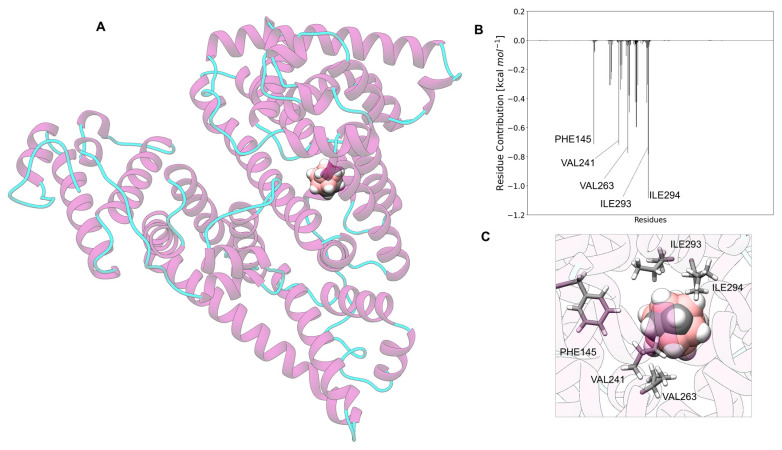
(**A**) A 3D representation of the carborane@afamin. (**B**) Per-residue decomposition energy. (**C**) Close-up of the most interacting protein residues with the ligand.

Afamin belongs to the albumin gene family, and is mainly expressed in the liver; its role in humans is related to the metabolism syndrome, i.e., the regulation of glucose in blood, obesity and other parameters [67]. Afamin shares ~35% similarity with HSA, and it bears similar binding pockets such as Sudlow’s site 1 and the deep cleft located in the center of the heart-shaped protein [67]. In these pockets, afamin is known to bind hydrophobic molecules, particularly vitamin E [67]. As represented in Figure 13A, carborane occupies Sudlow’s site 1 of afamin, interacting with Ile293 (−1.2 kcal mol^−1^), Ile294 (−0.9 kcal mol^−1^), Val263 (−0.8 kcal mol^−1^) and Val241 (−0.6 kcal mol^−1^) with hydrophobic interactions, and with Phe145 (−0.8 kcal mol^−1^) via BH-*π* interactions.

### 3.13. Analysis of the Nature of the Non-Covalent Interactions between the Amino Acids and the Carborane in the Protein-Binding Pockets

**Figure 14:**
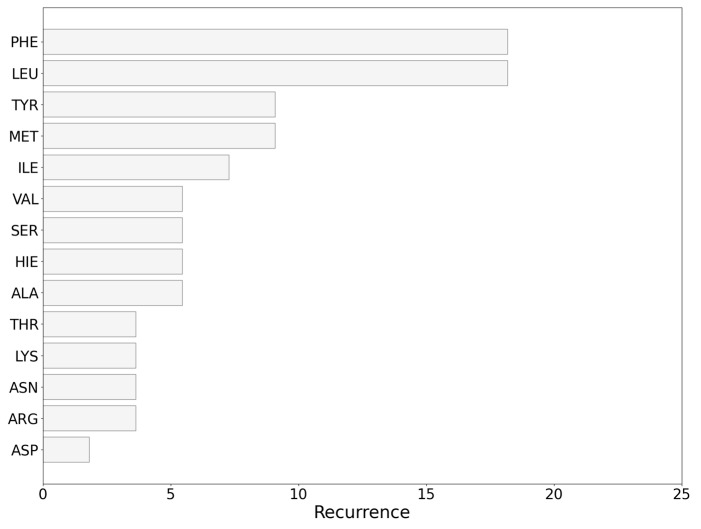
Recurrence (%) of amino acids in the binding pockets discussed in the present work.

The analysis of the most interacting amino acids (the five most interacting amino acids were selected for every protein considered) allows the identification of the most recurrent amino acids, responsible for the interaction of a protein with the carborane cage (Figure 14). 

The driving force in the recognition of the carborane in the protein-binding pockets is represented by:

(i) Hydrophobic interactions of the hydrophobic cage of the carborane with the aliphatic side chains of amino acids such as leucine (18%), methionine (9%), isoleucine (7%), valine (5%) and alanine (5%);
(ii) *B*H-π and *C*H-π interactions of the hydridic B-H groups and acidic C-H groups of the carborane with aromatic amino acids such as phenylalanine (18%), tyrosine (9%) and histidine (5%).

Other important contributions arise from the:

(iii) Formation of dihydrogen bonds with serine (5%), threonine (4%) and asparagine (4%), where serine and threonine use their hydroxylic moieties, while asparagine uses its amidic N-H to form the dihydrogen bonds with the hydridic B-H groups of the carborane;
(iv) Surfactant-like interactions with lysine (4%) and arginine (4%) which wrap the hydrophobic cage of the carborane with their aliphatic chains, while their hydrophilic moieties interact with water;
(v) Formation of classical hydrogen bonds with Asp (2.5%) where the carborane interacts with its acidic C-H groups with the carboxylate group of the amino acid.

## 4. Conclusions

A virtual screening approach allowed the identification of blood transport proteins as candidate carriers of carboranes. Human serum albumin (HSA) and transthyretin have already demonstrated their ability to bind carborane derivatives, but the structural characterization of their complexes has never been reported. Here, we identified the most probable binding sites for carborane in HSA, i.e., the FA5-binding pocket, Sudlow’s I site and the cleft region. In transthyretin, as suggested also by experimental data, the carborane occupies the T4-binding site. Hemoglobin showed a higher binding affinity for carboranes than transthyretin and human serum albumin (HSA). Myoglobin, ceruloplasmin, sex hormone-binding protein, lactoferrin, plasma retinol-binding protein, thyroxine-binding globulin, corticosteroid-binding globulin and afamin have a binding affinity comparable to HSA/transthyretin. The “blind identification” of HSA and transthyretin as carborane-binding proteins (second and third position in the ranking) vouches for the accuracy of the model and validates its predictions.

Carborane shows the tendency to bind in the protein pockets where hydrophobic molecules, such as fatty acids, heme, thyroxine, androgens and retinol, are usually recognized. The driving force for the carborane binding is represented by the formation of hydrophobic interactions with aliphatic amino acids (i.e., leucine, methionine) and *B*H-π and *C*H-π interactions with aromatic amino acids (i.e., phenylalanine, tyrosine). Dihydrogen bonds, classical hydrogen bonds and surfactant-like interactions also assist the binding. 

The blood transport proteins identified by the reverse docking protocol are potentially responsible to bind carborane upon their intravenous administration. These proteins can be also exploited, ex vivo, to develop boron neutron capture targeted therapy, following two different strategies: (i) the chemical conjugation of targeting (i.e., folate) and imaging (i.e., TRITC) tags on the surface of the protein to both improve the cell selectivity and promote the uptake of the carborane@protein hybrids in cancer cells, generating innovative protein-based theranostic platforms, and (ii) the selection of proteins that can be selectively uptaken by cancer cells via active targeting, such as albumin or transferrin, because their receptors are overexpressed in many cancer cells.

In addition, the dispersion of carboranes with specific proteins able to pass the BBB represents an opportunity for the development of a drug delivery system to target brain cancers with BNCT. Future works will be carried out to experimentally test the possibility to use the identified blood transport proteins as carborane carriers for BCNT. 

## Figures and Tables

**Table 1:** Structural database of blood transport protein structures used.

Protein	PDB/UNIPROT ID	Protein Concentration
Albumin	1N5U	42 mg/mL [74]
Afamin	6FAK	35 μg/mL [74]
α -fetoprotein	7YIM	20 ng/mL [75]
Cerulosplasmin	4ENZ	64.9 μg/mL [74]
Corticosteroid-binding globulin	2VDX	1.2 μg/mL [76]
Haptoglobulin	4X0L	1.1 mg/mL [74]
Hemoglobin	5HY8	41 μg/mL [74]
Hemopexin	P02790	257 μg/mL [74]
Lactotransferrin	1CB6	270 ng/mL [74]
Myoglobin	3RGK	210 ng/mL [76]
Plasma retinol-binding protein	5NU7	32 μg/mL [74]
Serotransferrin	6SOY	1.5 mg/mL [74]
Sex hormone-binding globulin	6PYF	260 ng/mL [76]
Thyroxine-binding globulin	2RIV	1.3 μg/mL [76]
Transthyretin	4QXV	109.6 μg/mL [74]
Vitamin D-binding protein	1KW2	62.36 μg/mL [74]

## Data Availability

All data in this study can be requested from the corresponding authors (tainah.marforio2@unibo.it (T.D.M.); matteo.calvaresi3@unibo.it (M.C.)).

## Supplementary Materials

The following supporting information can be downloaded at: https://www.mdpi.com/article/10.3390/nano13111770/s1, Figures S1–S15: RMSD of carborane@protein complexes.

## References

[1] Malouff T.D., Seneviratne D.S., Ebner D.K., Stross W.C., Waddle M.R., Trifiletti D.M., Krishnan S. (2021). Boron Neutron Capture Therapy: A Review of Clinical Applications. Front. Oncol..

[2] Murphy N., McCarthy E., Dwyer R., Farràs P. (2021). Boron Clusters as Breast Cancer Therapeutics. J. Inorg. Biochem..

[3] Nuez-Martinez M., Pinto C.I.G., Guerreiro J.F., Mendes F., Marques F., Muñoz-Juan A., Xavier J.A.M., Laromaine A., Bitonto V., Protti N. (2021). Cobaltabis(Dicarbollide) ([o-COSAN]−) as Multifunctional Chemotherapeutics: A Prospective Application in Boron Neutron Capture Therapy (BNCT) for Glioblastoma. Cancers.

[4] Messner K., Vuong B., Tranmer G.K. (2022). The Boron Advantage: The Evolution and Diversification of Boron’s Applications in Medicinal Chemistry. Pharmaceuticals.

[5] Ali F., Hosmane N.S., Zhu Y. (2020). Boron Chemistry for Medical Applications. Molecules.

[6] Cheng X., Li F., Liang L. (2022). Boron Neutron Capture Therapy: Clinical Application and Research Progress. Curr. Oncol..

[7] Lamba M., Goswami A., Bandyopadhyay A. (2021). A Periodic Development of BPA and BSH Based Derivatives in Boron Neutron Capture Therapy (BNCT). Chem. Commun..

[8] Zharkov D.O., Yudkina A.V., Riesebeck T., Loshchenova P.S., Mostovich E.A., Dianov G.L. (2021). Boron-Containing Nucleosides as Tools for Boron-Neutron Capture Therapy. Am. J. Cancer Res..

[9] Gruzdev D.A., Levit G.L., Krasnov V.P., Charushin V.N. (2021). Carborane-Containing Amino Acids and Peptides: Synthesis, Properties and Applications. Coord. Chem. Rev..

[10] Alamón C., Dávila B., García M.F., Sánchez C., Kovacs M., Trias E., Barbeito L., Gabay M., Zeineh N., Gavish M. (2020). Sunitinib-Containing Carborane Pharmacophore with the Ability to Inhibit Tyrosine Kinases Receptors FLT3, KIT and PDGFR-β, Exhibits Powerful In Vivo Anti-Glioblastoma Activity. Cancers.

[11] Barth R.F., Yang W., Al-Madhoun A.S., Johnsamuel J., Byun Y., Chandra S., Smith D.R., Tjarks W., Eriksson S. (2004). Boron-Containing Nucleosides as Potential Delivery Agents for Neutron Capture Therapy of Brain Tumors. Cancer Res..

[12] Leśnikowski Z.J., Paradowska E., Olejniczak A.B., Studzińska M., Seekamp P., Schüßler U., Gabel D., Schinazi R.F., Plešek J. (2005). Towards New Boron Carriers for Boron Neutron Capture Therapy: Metallacarboranes and Their Nucleoside Conjugates. Bioorganic Med. Chem..

[13] Smilowitz H.M., Slatkin D.N., Micca P.L., Miura M. (2013). Microlocalization of Lipophilic Porphyrins: Non-Toxic Enhancers of Boron Neutron-Capture Therapy. Int. J. Radiat. Biol..

[14] Miura M., Morris G.M., Hopewell J.W., Micca P.L., Makar M.S., Nawrocky M.M., Renner M.W. (2014). Enhancement of the Radiation Response of EMT-6 Tumours by a Copper Octabromotetracarboranylphenylporphyrin. Br. J. Radiol..

[15] Kawabata S., Yang W., Barth R.F., Wu G., Huo T., Binns P.J., Riley K.J., Ongayi O., Gottumukkala V., Vicente M.G.H. (2011). Convection Enhanced Delivery of Carboranylporphyrins for Neutron Capture Therapy of Brain Tumors. J. Neurooncol..

[16] Hu K., Yang Z., Zhang L., Xie L., Wang L., Xu H., Josephson L., Liang S.H., Zhang M.R. (2020). Boron Agents for Neutron Capture Therapy. Coord. Chem. Rev..

[17] Imperio D., Panza L. (2022). Sweet Boron: Boron-Containing Sugar Derivatives as Potential Agents for Boron Neutron Capture Therapy. Symmetry.

[18] Schwarze B., Gozzi M., Zilberfain C., Rüdiger J., Birkemeyer C., Estrela-Lopis I., Hey-Hawkins E. (2020). Nanoparticle-Based Formulation of Metallacarboranes with Bovine Serum Albumin for Application in Cell Cultures. J. Nanoparticle Res..

[19] Kawasaki R., Sasaki Y., Akiyoshi K. (2017). Self-Assembled Nanogels of Carborane-Bearing Polysaccharides for Boron Neutron Capture Therapy. Chem. Lett..

[20] Silva W.M., Ribeiro H., Taha-Tijerina J.J. (2021). Potential Production of Theranostic Boron Nitride Nanotubes (^64^Cu-BNNTs) Radiolabeled by Neutron Capture. Nanomaterials.

[21] Chiang C.W., Chien Y.C., Yu W.J., Ho C.Y., Wang C.Y., Wang T.W., Chiang C.S., Keng P.Y. (2021). Polymer-Coated Nanoparticles for Therapeutic and Diagnostic Non-^10^B Enriched Polymer-Coated Boron Carbon Oxynitride (BCNO) Nanoparticles as Potent BNCT Drug. Nanomaterials.

[22] Pitto-Barry A. (2021). Polymers and Boron Neutron Capture Therapy (BNCT): A Potent Combination. Polym. Chem..

[23] Takeuchi I., Kishi N., Shiokawa K., Uchiro H., Makino K. (2018). Polyborane Encapsulated Liposomes Prepared Using PH Gradient and Reverse-Phase Evaporation for Boron Neutron Capture Therapy: Biodistribution in Tumor-Bearing Mice. Colloid Polym. Sci..

[24] Xiong H., Wei X., Zhou D., Qi Y., Xie Z., Chen X., Jing X., Huang Y. (2016). Amphiphilic Polycarbonates from Carborane-Installed Cyclic Carbonates as Potential Agents for Boron Neutron Capture Therapy. Bioconjugate Chem..

[25] Lee W., Sarkar S., Ahn H., Kim J.Y., Lee Y.J., Chang Y., Yoo J. (2020). PEGylated Liposome Encapsulating Nido-Carborane Showed Significant Tumor Suppression in Boron Neutron Capture Therapy (BNCT). Biochem. Biophys. Res. Commun..

[26] Tsygankova A.R., Gruzdev D.A., Kanygin V.V., Guselnikova T.Y., Telegina A.A., Kasatova A.I., Kichigin A.I., Levit G.L., Mechetina L.V., Mukhamadiyarov R.A. (2021). Liposomes Loaded with Lipophilic Derivative of Closo-Carborane as a Potential Boron Delivery System for Boron Neutron Capture Therapy of Tumors. Mendeleev. Commun..

[27] Singh B., Kaur G., Singh P., Singh K., Kumar B., Vij A., Kumar M., Bala R., Meena R., Singh A. (2016). Nanostructured Boron Nitride With High Water Dispersibility For Boron Neutron Capture Therapy. Sci. Rep..

[28] Ailuno G., Balboni A., Caviglioli G., Lai F., Barbieri F., Dellacasagrande I., Florio T., Baldassari S. (2022). Boron Vehiculating Nanosystems for Neutron Capture Therapy in Cancer Treatment. Cells.

[29] Sumitani S., Nagasaki Y. (2012). Boron Neutron Capture Therapy Assisted by Boron-Conjugated Nanoparticles. Polym. J..

[30] Dukenbayev K., Korolkov I.V., Tishkevich D.I., Kozlovskiy A.L., Trukhanov S.V., Gorin Y.G., Shumskaya E.E., Kaniukov E.Y., Vinnik D.A., Zdorovets M.V. (2019). Fe_3_O_4_ Nanoparticles for Complex Targeted Delivery and Boron Neutron Capture Therapy. Nanomaterials.

[31] Ferreira T.H., Miranda M.C., Rocha Z., Leal A.S., Gomes D.A., Sousa E.M.B. (2017). An Assessment of the Potential Use of BNNTs for Boron Neutron Capture Therapy. Nanomaterials.

[32] Oleshkevich E., Morancho A., Saha A., Galenkamp K.M.O., Grayston A., Crich S.G., Alberti D., Protti N., Comella J.X., Teixidor F. (2019). Combining Magnetic Nanoparticles and Icosahedral Boron Clusters in Biocompatible Inorganic Nanohybrids for Cancer Therapy. Nanomedicine.

[33] Ferrer-Ugalde A., Sandoval S., Pulagam K.R., Muñoz-Juan A., Laromaine A., Llop J., Tobias G., Núñez R. (2021). Radiolabeled Cobaltabis(Dicarbollide) Anion-Graphene Oxide Nanocomposites for in Vivo Bioimaging and Boron Delivery. ACS Appl. Nano Mater..

[34] Yan J., Yang W., Zhang Q., Yan Y. (2020). Introducing Borane Clusters into Polymeric Frameworks: Architecture, Synthesis, and Applications. Chem. Commun..

[35] Marfavi A., Kavianpour P., Rendina L.M. (2022). Carboranes in Drug Discovery, Chemical Biology and Molecular Imaging. Nat. Rev. Chem..

[36] Poater J., Viñas C., Bennour I., Escayola S., Solà M., Teixidor F. (2020). Too Persistent to Give Up: Aromaticity in Boron Clusters Survives Radical Structural Changes. J. Am. Chem. Soc..

[37] Farràs P., Juárez-Pérez E.J., Lepšík M., Luque R., Núñez R. (2012). Metallacarboranes and Their Interactions: Theoretical Insights and Their Applicability. Chem. Soc. Rev..

[38] Issa F., Kassiou M., Rendina L.M. (2011). Boron in Drug Discovery: Carboranes as Unique Pharmacophores in Biologically Active Compounds. Chem. Rev..

[39] Chen Y., Du F., Tang L., Xu J., Zhao Y., Wu X., Li M., Shen J., Wen Q., Cho C.H. (2022). Carboranes as Unique Pharmacophores in Antitumor Medicinal Chemistry. Mol. Ther. Oncolytics.

[40] Barry N.P.E., Sadler P.J. (2012). Dicarba-Closo-Dodecarborane-Containing Half-Sandwich Complexes of Ruthenium, Osmium, Rhodium and Iridium: Biological Relevance and Synthetic Strategies. Chem. Soc. Rev..

[41] Fink K., Uchman M. (2021). Boron Cluster Compounds as New Chemical Leads for Antimicrobial Therapy. Coord. Chem. Rev..

[42] Stockmann P., Gozzi M., Kuhnert R., Sárosi M.B., Hey-Hawkins E. (2019). New Keys for Old Locks: Carborane-Containing Drugs as Platforms for Mechanism-Based Therapies. Chem. Soc. Rev..

[43] Fithroni A.B., Kobayashi K., Uji H., Ishimoto M., Akehi M., Ohtsuki T., Matsuura E. (2022). Novel Self-Forming Nanosized DDS Particles for BNCT: Utilizing A Hydrophobic Boron Cluster and Its Molecular Glue Effect. Cells.

[44] Takeuchi I., Nomura K., Makino K. (2017). Hydrophobic Boron Compound-Loaded Poly(l-Lactide-Co-Glycolide) Nanoparticles for Boron Neutron Capture Therapy. Colloids Surf. B Biointerfaces.

[45] Alberti D., Protti N., Toppino A., Deagostino A., Lanzardo S., Bortolussi S., Altieri S., Voena C., Chiarle R., Crich S.G. (2015). A Theranostic Approach Based on the Use of a Dual Boron/Gd Agent to Improve the Efficacy of Boron Neutron Capture Therapy in the Lung Cancer Treatment. Nanomedicine.

[46] Heber E.M., Kueffer P.J., Lee M.W., Hawthorne M.F., Garabalino M.A., Molinari A.J., Nigg D.W., Bauer W., Hughes A.M., Pozzi E.C.C. (2012). Boron Delivery with Liposomes for Boron Neutron Capture Therapy (BNCT): Biodistribution Studies in an Experimental Model of Oral Cancer Demonstrating Therapeutic Potential. Radiat. Environ. Biophys..

[47] Zhang T., Xu D., Yi Y., Wang Y., Cui Z., Chen X., Ma Q., Song F., Zhu B., Zhao Z. (2022). Chitosan-Lactobionic Acid-Thioctic Acid-Modified Hollow Mesoporous Silica Composite Loaded with Carborane for Boron Neutron Capture Therapy of Hepatocellular Carcinoma. Mater. Des..

[48] Rak J., Kaplánek R., Král V. (2010). Solubilization and Deaggregation of Cobalt Bis(Dicarbollide) Derivatives in Water by Biocompatible Excipients. Bioorganic Med. Chem. Lett..

[49] Rak J., Jakubek M., Kaplánek R., Matějíček P., Král V. (2011). Cobalt Bis(Dicarbollide) Derivatives: Solubilization and Self-Assembly Suppression. Eur. J. Med. Chem..

[50] Rak J., Dejlová B., Lampová H., Kaplánek R., Matějíček P., Cígler P., Král V. (2013). On the Solubility and Lipophilicity of Metallacarborane Pharmacophores. Mol. Pharm..

[51] Marconi A., Giugliano G., Di Giosia M., Marforio T.D., Trivini M., Turrini E., Fimognari C., Zerbetto F., Mattioli E.J., Calvaresi M. (2023). Identification of Blood Transport Proteins to Carry Temoporfin: A Domino Approach from Virtual Screening to Synthesis and In Vitro PDT Testing. Pharmaceutics.

[52] Cantelli A., Malferrari M., Mattioli E.J., Marconi A., Mirra G., Soldà A., Marforio T.D., Zerbetto F., Rapino S., Di Giosia M. (2022). Enhanced Uptake and Phototoxicity of C_60_@albumin Hybrids by Folate Bioconjugation. Nanomaterials.

[53] Mattioli E.J., Ulfo L., Marconi A., Pellicioni V., Costantini P.E., Marforio T.D., Di Giosia M., Danielli A., Fimognari C., Turrini E. (2023). Carrying Temoporfin with Human Serum Albumin: A New Perspective for Photodynamic Application in Head and Neck Cancer. Biomolecules.

[54] Marconi A., Mattioli E.J., Ingargiola F., Giugliano G., Marforio T.D., Prodi L., Di Giosia M., Calvaresi M. (2023). Dissecting the Interactions between Chlorin E6 and Human Serum Albumin. Molecules.

[55] Calvaresi M., Zerbetto F. (2011). In Silico Carborane Docking to Proteins and Potential Drug Targets. J. Chem. Inf. Model..

[56] Di Giosia M., Zerbetto F., Calvaresi M. (2021). Incorporation of Molecular Nanoparticles inside Proteins: The Trojan Horse Approach in Theranostics. Acc. Mater. Res..

[57] Frontera A., Bauzá A. (2019). Closo -Carboranes as Dual CH⋯π and BH⋯π Donors: Theoretical Study and Biological Significance. Phys. Chem. Chem. Phys..

[58] Zou W., Zhang X., Dai H., Yan H., Cremer D., Kraka E. (2018). Description of an Unusual Hydrogen Bond between Carborane and a Phenyl Group. J. Organomet. Chem..

[59] Saha B., Sharma H., Bhattacharyya P.K. (2019). Nonclassical B-H_b_⋯π Interaction in Diborane⋯localized-π Sandwiches: A DFT-D3 Study. Int. J. Quantum. Chem..

[60] Fanfrlík J., Brynda J., Kugler M., Lepšik M., Pospísilová K., Holub J., Hnyk D., Nekvinda J., Gruner B., Rezacova P. (2023). B-H⋯π and C-H⋯π Interactions in Protein-Ligand Complexes: Carbonic Anhydrase II Inhibition by Carborane Sulfonamides. Phys. Chem. Chem. Phys..

[61] Zhang X., Dai H., Yan H., Zou W., Cremer D. (2016). B−H···π Interaction: A New Type of Nonclassical Hydrogen Bonding. J. Am. Chem. Soc..

[62] Di Giosia M., Soldà A., Seeger M., Cantelli A., Arnesano F., Nardella M.I., Mangini V., Valle F., Montalti M., Zerbetto F. (2021). A Bio-Conjugated Fullerene as a Subcellular-Targeted and Multifaceted Phototheranostic Agent. Adv. Funct. Mater..

[63] Cantelli A., Malferrari M., Solda A., Simonetti G., Forni S., Toscanella E., Mattioli E.J., Zerbetto F., Zanelli A., Di Giosia M. (2021). Human Serum Albumin−oligothiophene Bioconjugate: A Phototheranostic Platform for Localized Killing of Cancer Cells by Precise Light Activation. JACS Au.

[64] Zhang N., Mei K., Guan P., Hu X., Zhao Y., Zhang N., Mei K., Guan P., Hu X., Zhao Y.L. (2020). Protein-Based Artificial Nanosystems in Cancer Therapy. Small.

[65] Liang K., Chen H. (2020). Protein-Based Nanoplatforms for Tumor Imaging and Therapy. Wiley Interdiscip. Rev. Nanomed. Nanobiotechnol..

[66] Gou Y., Miao D., Zhou M., Wang L., Zhou H., Su G. (2018). Bio-Inspired Protein-Based Nanoformulations for Cancer Theranostics. Front. Pharmacol..

[67] Schaller J., Gerber S., Kämpfer U., Lejon S., Trachsel C. (2008). Human Blood Plasma Proteins: Structure and Function.

[68] Greco G., Ulfo L., Turrini E., Marconi A., Costantini P.E., Marforio T.D., Mattioli E.J., Di Giosia M., Danielli A., Fimognari C. (2023). Light-Enhanced Cytotoxicity of Doxorubicin by Photoactivation. Cells.

[69] Shan L., Hao Y., Wang S., Korotcov A., Zhang R., Wang T., Califano J., Gu X., Sridhar R., Bhujwalla Z.M. (2008). Visualizing Head and Neck Tumors in Vivo Using Near-Infrared Fluorescent Transferrin Conjugate. Mol. Imaging.

[70] Desai N., Trieu V., Damascelli B., Soon-Shiong P. (2009). SPARC Expression Correlates with Tumor Response to Albumin-Bound Paclitaxel in Head and Neck Cancer Patients. Transl. Oncol..

[71] Calabrese G., Daou A., Barbu E., Tsibouklis J. (2018). Towards Carborane-Functionalised Structures for the Treatment of Brain Cancer. Drug Discov. Today.

[72] Berman H.M., Westbrook J., Feng Z., Gilliland G., Bhat T.N., Weissig H., Shindyalov I.N., Bourne P.E. (2000). The Protein Data Bank. Nucleic Acids Res..

[73] Jumper J., Evans R., Pritzel A., Green T., Figurnov M., Ronneberger O., Tunyasuvunakool K., Bates R., Žídek A., Potapenko A. (2021). Highly Accurate Protein Structure Prediction with AlphaFold. Nature.

[74] Percy A.J., Chambers A.G., Smith D.S., Borchers C.H. (2013). Standardized Protocols for Quality Control of MRM-Based Plasma Proteomic Workflows. J. Proteome Res..

[75] Polanski M., Anderson N.L. (2006). A List of Candidate Cancer Biomarkers for Targeted Proteomics. Biomark Insights.

[76] Farrah T., Deutsch E.W., Omenn G.S., Campbell D.S., Sun Z., Bletz J.A., Mallick P., Katz J.E., Malmström J., Ossola R. (2011). A High-Confidence Human Plasma Proteome Reference Set with Estimated Concentrations in PeptideAtlas. Mol. Cell. Proteom..

[77] Schneidman-Duhovny D., Inbar Y., Polak V., Shatsky M., Halperin I., Benyamini H., Barzilai A., Dror O., Haspel N., Nussinov R. (2003). Taking Geometry to Its Edge: Fast Unbound Rigid (and Hinge-Bent) Docking. Proteins Struct. Funct. Genet..

[78] Calvaresi M., Zerbetto F. (2010). Baiting Proteins with C60. ACS Nano.

[79] Calvaresi M., Zerbetto F. (2011). Fullerene Sorting Proteins. Nanoscale.

[80] Ahmed L., Rasulev B., Kar S., Krupa P., Mozolewska M.A., Leszczynski J. (2017). Inhibitors or Toxins? Large Library Target-Specific Screening of Fullerene-Based Nanoparticles for Drug Design Purpose. Nanoscale.

[81] di Giosia M., Valle F., Cantelli A., Bottoni A., Zerbetto F., Calvaresi M. (2018). C60 Bioconjugation with Proteins: Towards a Palette of Carriers for All PH Ranges. Materials.

[82] Calvaresi M., Furini S., Domene C., Bottoni A., Zerbetto F. (2015). Blocking the Passage: C_60_ Geometrically Clogs K+ Channels. ACS Nano.

[83] Calvaresi M., Arnesano F., Bonacchi S., Bottoni A., Calò V., Conte S., Falini G., Fermani S., Losacco M., Montalti M. (2014). C_60_@Lysozyme: Direct Observation by Nuclear Magnetic Resonance of a 1:1 Fullerene Protein Adduct. ACS Nano.

[84] Calvaresi M., Bottoni A., Zerbetto F. (2015). Thermodynamics of Binding between Proteins and Carbon Nanoparticles: The Case of C60@Lysozyme. J. Phys. Chem. C.

[85] Marforio T.D., Mattioli E.J., Zerbetto F., Calvaresi M. (2022). Fullerenes against COVID-19: Repurposing C_60_ and C_70_ to Clog the Active Site of SARS-CoV-2 Protease. Molecules.

[86] Malarz K., Korzuch J., Marforio T.D., Balin K., Calvaresi M., Mrozek-Wilczkiewicz A., Musiol R., Serda M. (2023). Identification and Biological Evaluation of a Water-Soluble Fullerene Nanomaterial as BTK Kinase Inhibitor. Int. J. Nanomed..

[87] Di Giosia M., Valle F., Cantelli A., Bottoni A., Zerbetto F., Fasoli E., Calvaresi M. (2019). Identification and Preparation of Stable Water Dispersions of Protein—Carbon Nanotube Hybrids and Efficient Design of New Functional Materials. Carbon.

[88] Berto M., Di Giosia M., Giordani M., Sensi M., Valle F., Alessandrini A., Menozzi C., Cantelli A., Gazzadi G.C., Zerbetto F. (2021). Green Fabrication of (6,5)Carbon Nanotube/Protein Transistor Endowed with Specific Recognition. Adv. Electron. Mater..

[89] Di Giosia M., Marforio T.D., Cantelli A., Valle F., Zerbetto F., Su Q., Wang H., Calvaresi M. (2020). Inhibition of α-Chymotrypsin by Pristine Single-Wall Carbon Nanotubes: Clogging up the Active Site. J. Colloid. Interface Sci..

[90] Calvaresi M., Hoefinger S., Zerbetto F. (2012). Probing the Structure of Lysozyme–Carbon-Nanotube Hybrids with Molecular Dynamics. Chem. A Eur. J..

[91] Wang J., Wolf R.M., Caldwell J.W., Kollman P.A., Case D.A. (2004). Development and Testing of a General Amber Force Field. J. Comput. Chem..

[92] Sárosi M.B., Lybrand T.P. (2018). Molecular Dynamics Simulation of Cyclooxygenase-2 Complexes with Indomethacin Closo-Carborane Analogs. J. Chem. Inf. Model..

[93] Roe D.R., Cheatham T.E. (2013). PTRAJ and CPPTRAJ: Software for Processing and Analysis of Molecular Dynamics Trajectory Data. J. Chem. Theory Comput..

[94] Miller B.R., McGee T.D., Swails J.M., Homeyer N., Gohlke H., Roitberg A.E. (2012). MMPBSA.Py: An Efficient Program for End-State Free Energy Calculations. J. Chem. Theory Comput..

[95] Hawkins G.D., Cramer C.J., Truhlar D.G. (1996). Parametrized Models of Aqueous Free Energies of Solvation Based on Pairwise Descreening of Solute Atomic Charges from a Dielectric Medium. J. Phys. Chem..

[96] Hawkins G.D., Cramer C.J., Truhlar D.G. (1995). Pairwise Solute Descreening of Solute Charges from a Dielectric Medium. Chem. Phys. Lett..

[97] Ol’shevskaya V.A., Nikitina R.G., Savchenko A.N., Malshakova M.V., Vinogradov A.M., Golovina G.V., Belykh D.V., Kutchin A.V., Kaplan M.A., Kalinin V.N. (2009). Novel Boronated Chlorin E6-Based Photosensitizers: Synthesis, Binding to Albumin and Antitumour Efficacy. Bioorganic Med. Chem..

[98] Goszczyński T.M., Fink K., Kowalski K., Leśnikowski Z.J., Boratyński J. (2017). Interactions of Boron Clusters and Their Derivatives with Serum Albumin. Sci. Rep..

[99] Jena B.B., Satish L., Mahanta C.S., Swain B.R., Sahoo H., Dash B.P., Satapathy R. (2019). Interaction of Carborane-Appended Trimer with Bovine Serum Albumin: A Spectroscopic Investigation. Inorg. Chim. Acta.

[100] Fuentes I., Pujols J., Viñas C., Ventura S., Teixidor F. (2019). Dual Binding Mode of Metallacarborane Produces a Robust Shield on Proteins. Chem. A Eur. J..

[101] Julius R.L., Farha O.K., Chiang J., Perry L.J., Hawthorne M.F. (2007). Synthesis and Evaluation of Transthyretin Amyloidosis Inhibitors Containing Carborane Pharmacophores. Proc. Natl. Acad. Sci. USA.

