# Supplementary material for: Exploiting Blood Transport Proteins as Carborane Supramolecular Vehicles for Boron Neutron Capture Therapy
Source: Nanomaterials (Basel). 2023 May 31;13(11):1770. doi: 10.3390/nano13111770 (PMC10254601; doi:10.3390/nano13111770)
Supplement: Supplementary file 1 [file nanomaterials-13-01770-s001.zip › nanomaterials-2386494-supplementary.pdf]

Supplementary Materials

Exploiting Blood Transport Proteins as Carborane Supramolecular Vehicles for Boron Neutron Capture Therapy

Tainah Dorina Marforio [1,*], Edoardo Jun Mattioli [1], Francesco Zerbetto [1], and Matteo Calvaresi [1,*]

[1] Dipartimento di Chimica "Giacomo Ciamician", Alma Mater Studiorum-Università di Bologna, Via Francesco Selmi 2, 40126 Bologna, Italy

* Correspondence: tainah.marforio2@unibo.it (T.D.M.); matteo.calvaresi3@unibo.it (M.C.).

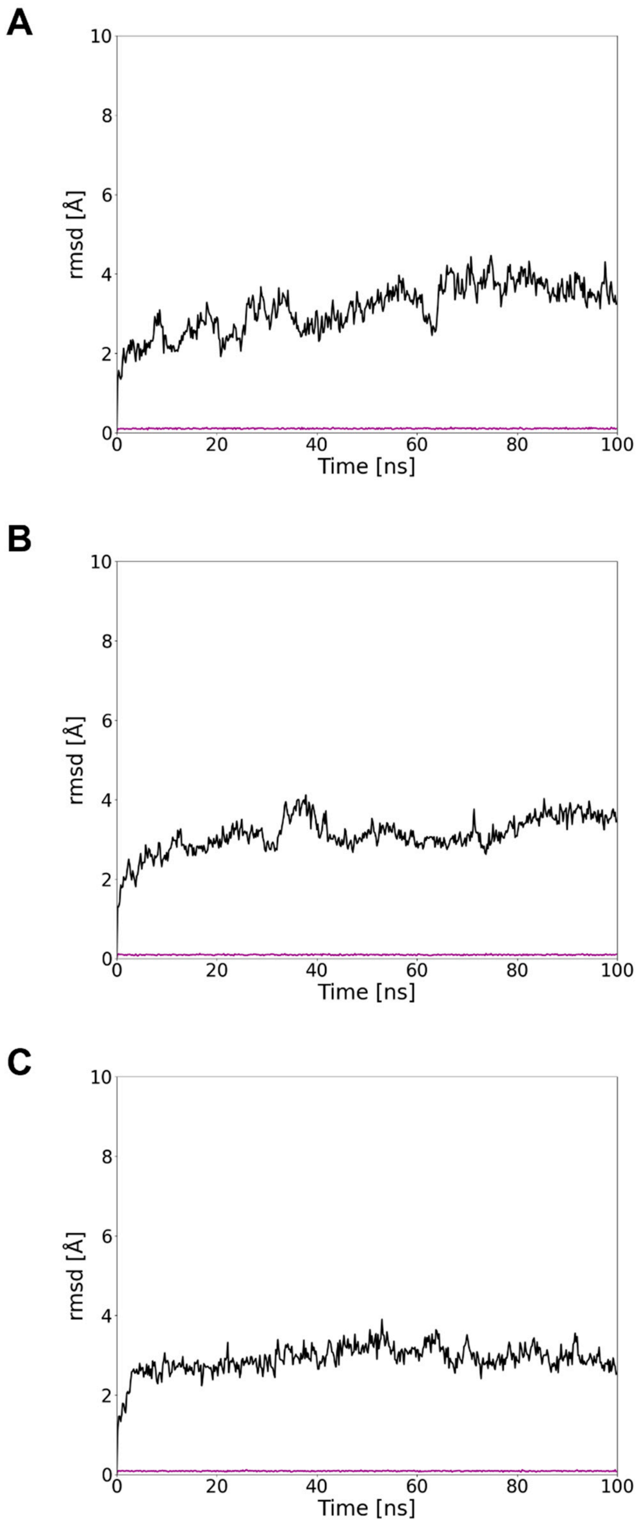

Figure S1. RMSD of carborane@HSA complexes in the A) FA5, B) Sudlow's I (FA7) and C) cleft binding sites. Purple line refers to carborane, while black line to the protein.

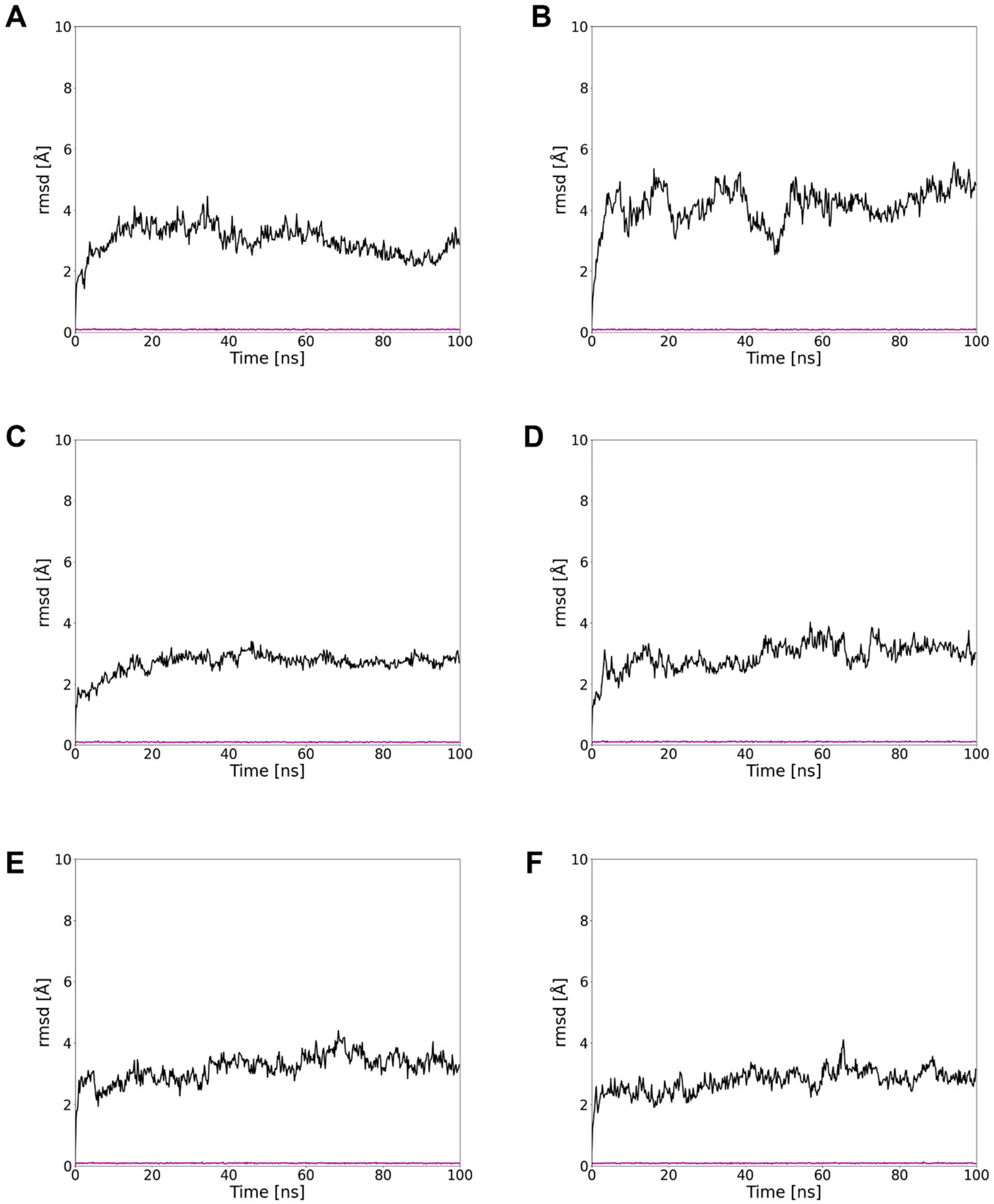

Figure S2. RMSD of carborane@HSA complexes in the A) FA2, B) cleft (bottom), C) NewIA, D) FA 3,4, E) FA6 and F) heme binding sites. Purple line refers to carborane, while black line to the protein

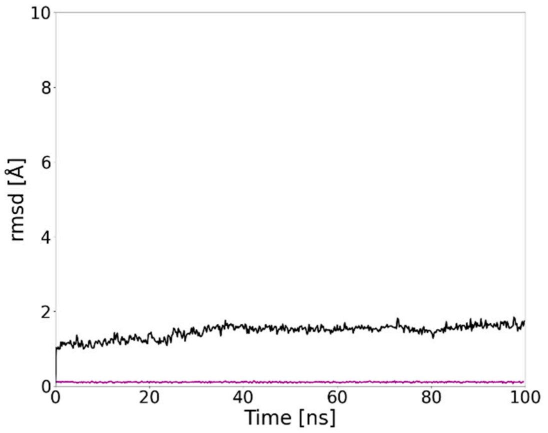

Figure S3. RMSD of carborane@transthyretin complex. Purple line refers to carborane, while black line to the protein.

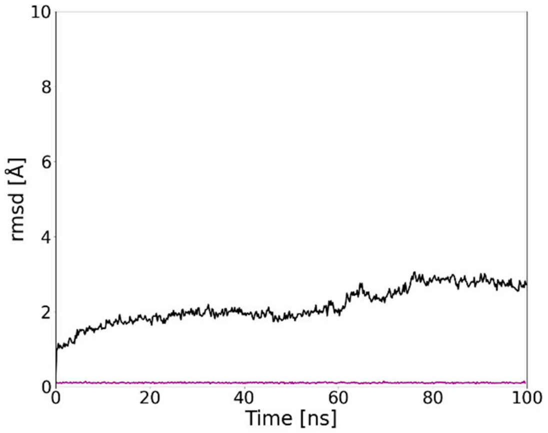

Figure S4. RMSD of carborane@hemoglobin complex. Purple line refers to carborane, while black line to the protein.

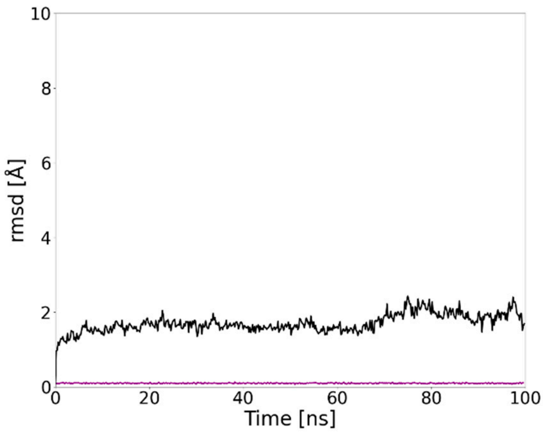

Figure S5. RMSD of carborane@myoglobin complex. Purple line refers to carborane, while black line to the protein.

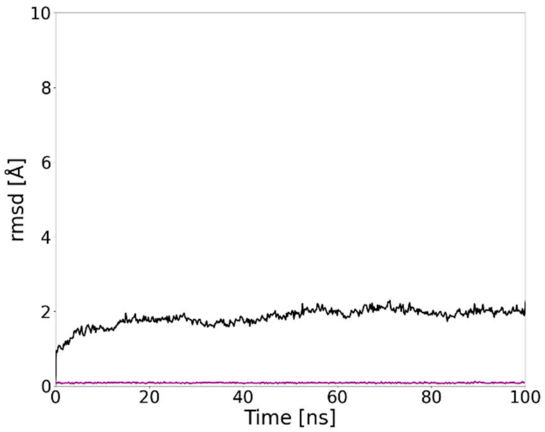

Figure S6. RMSD of carborane@ceruloplasmin complex. Purple line refers to carborane, while black line to the protein.

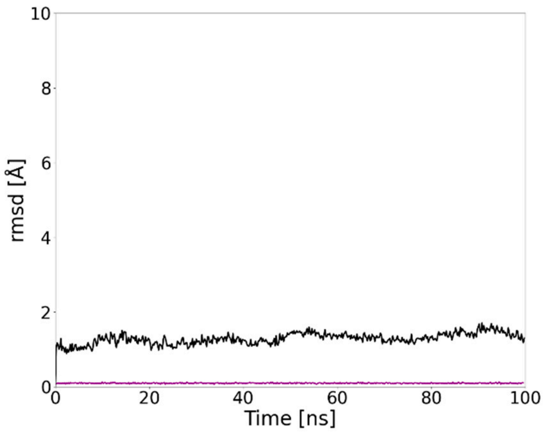

Figure S7. RMSD of carborane@sex hormone-binding globulin complex. Purple line refers to carborane, while black line to the protein

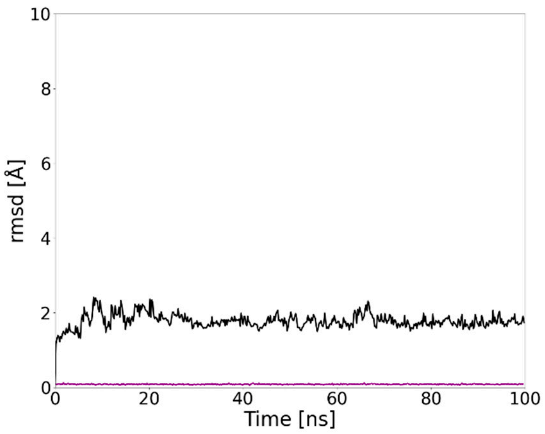

Figure S8. RMSD of carborane@lactoferrin complex. Purple line refers to carborane, while black line to the protein

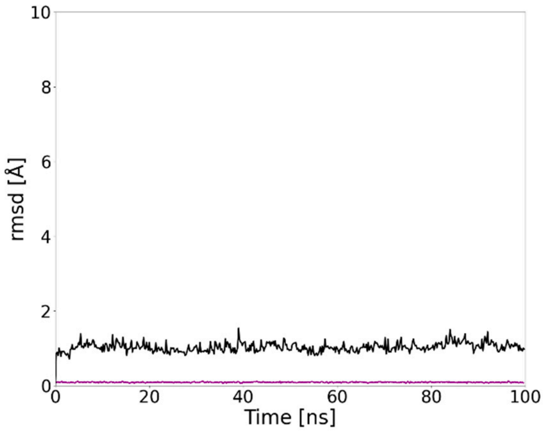

Figure S9. RMSD of carborane@plasma retinol-binding protein complex. Purple line refers to carborane, while black line to the protein.

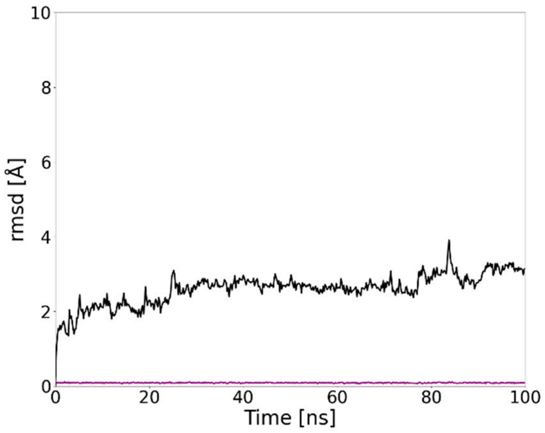

Figure S10. RMSD of carborane@thyroxyne-binding protein complex. Purple line refers to carborane, while black line to the protein.

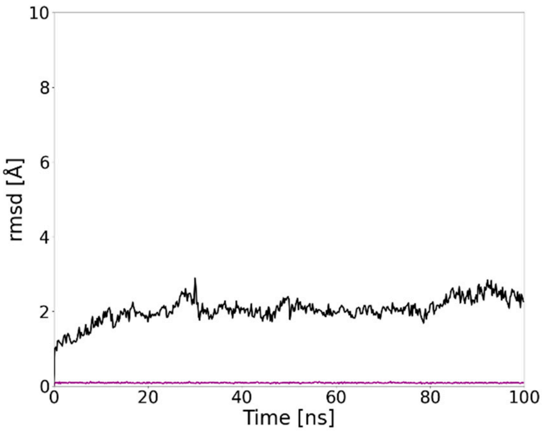

Figure S11. RMSD of carborane@corticosteroid-binding protein complex. Purple line refers to carborane, while black line to the protein.

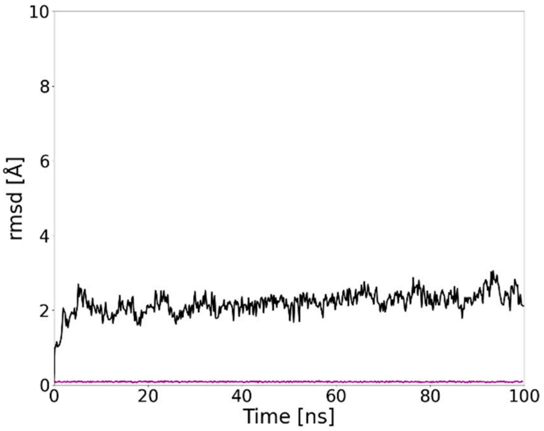

Figure S12. RMSD of carborane@afamin complex. Purple line refers to carborane, while black line to the protein.

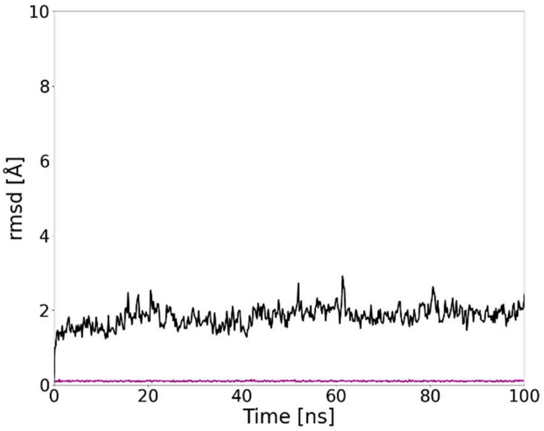

Figure S13. RMSD of carborane@serotransferrin complex. Purple line refers to carborane, while black line to the protein.

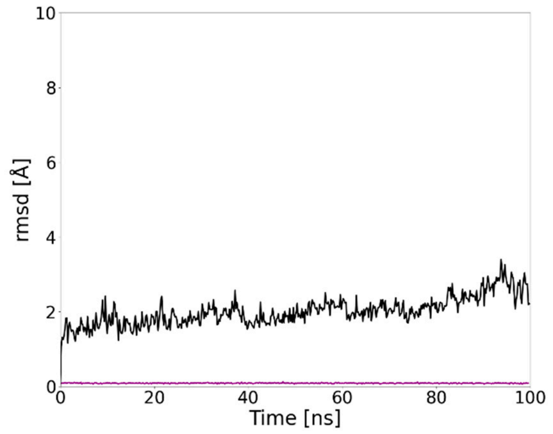

Figure S14. RMSD of carborane@vitamin D-binding protein complex. Purple line refers to carborane, while black line to the protein.

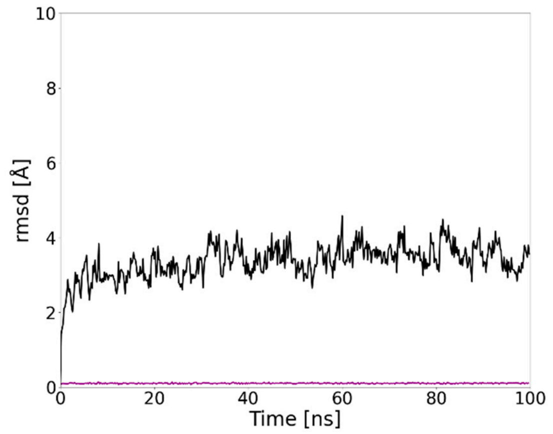

Figure S15. RMSD of carborane@alpha-fetoprotein complex. Purple line refers to carborane, while black line to the protein.
